# For or against Adjuvant Trastuzumab for pT1a-bN0M0 Breast Cancer Patients with HER2-Positive Tumors: A Meta-Analysis of Published Literatures

**DOI:** 10.1371/journal.pone.0083646

**Published:** 2014-01-02

**Authors:** Qiong Zhou, Wenjin Yin, Yueyao Du, Jinsong Lu

**Affiliations:** 1 Department of Breast Surgery, Fudan University Shanghai Cancer Center; Department of Oncology, Shanghai Medical College, Fudan University, Shanghai, China; 2 Department of Breast Surgery, Renji Hospital, School of Medicine, Shanghai Jiaotong University, Shanghai, China; Health Canada and University of Ottawa, Canada

## Abstract

**Background:**

Although the prognosis of patients with small (≤1cm) tumors is generally favorable, emerging data suggests that biological behavior varies between intrinsic subtypes in such patients. Furthermore, it still remains unclear whether HER2-positive pT1a-bN0M0 patients could benefit from adjuvant trastuzumab. For further evaluation, we sought to conduct a meta-analysis so as to get a better understanding of the prognosis for HER2-positive pT1a-bN0M0 patients and their survival benefit from adjuvant trastuzumab, accordingly, offering the implications for current practice.

**Methods:**

The PubMed database, the online proceedings of the American Society of Clinical Oncology (ASCO) Annual Meetings, the online proceedings of the San Antonio Breast Cancer Symposium, and the CD proceedings of the International St. Gallen Breast Cancer Conference were searched for all relevant studies published before September 2012. Relative risks (RRs) were used to compare the prognosis of different intrinsic subtypes for pT1a-bN0M0 breast cancer. Analyses were also performed to estimate the association between adjuvant trastuzumab and various survival outcomes.

**Results:**

With eight eligible studies identified, this meta-analysis demonstrated a deleterious effect of HER2+ phenotype on disease-free survival (DFS; RR = 3.677, 95% CI 2.606–5.189, *p* <0.001) and distant disease-free survival (DDFS; RR = 3.824, 95% CI 2.249–6.501, *p*<0.001) as compared to HR+/HER2- subgroup. However, significant difference failed to be achieved in terms of any endpoint between HER2+ and triple negative breast cancer (TNBC). Besides, a marked improvement in DFS was observed with the addition of trastuzumab for HER2-positive pT1a-bN0M0 patients (RR = 0.323, 95% CI 0.191–0.547, *p*<0.001).

**Conclusion:**

This meta-analysis clarifies that intrinsic subtypes might be a reliable marker to predict the prognosis in pT1a-bN0M0 breast cancer. Besides, even for such early stage HER2-positive patients, adjuvant trastuzumab might bring significant survival benefit.

## Introduction

The prognosis of patients with small, infracentimetric breast cancer that are regional lymph node (including ipsilateral axillary, internal mammary, infraclavicular and supraclavicular lymph nodes) negative (pT1a-bN0M0) is generally favorable. Nevertheless, relapses and deaths do occur in such breast cancer patients, especially for those with human epidermal growth factor receptor 2 (HER2)-positive tumors. HER2 positivity is defined as either the overexpression of HER2 protein (immunohistochemistry 3+) or the amplification of *HER2/neu* gene (*in situ* hybridization positive) according to the American Society of Clinical Oncology (ASCO)/College of American Pathologists (CAP) Clinical Practice Guideline [1]. Manifold retrospective studies have demonstrated that HER2-positive status could help distinguish a subset of patients with inferior outcomes as compared with the counterparts [2]–[5]. Furthermore, a recent meta-analysis has provided further support for the prognostic role of HER2 in pT1a-bN0M0 patients [6]. With the increasing recognition that breast cancer is a heterogeneous disease classified as different subtypes with distinctive biology, however, it seems far from enough to guide risk allocation merely according to HER2 status in pT1a-bN0M0 breast cancer. Over recent years, the growing attention has been devoted to the predictive value of intrinsic subtypes in clinical outcomes for such patients [2], [4], [7]–[11]. Unfortunately, various definitions and conflicting results provide an unclear picture. In most studies, patients were divided as per hormone receptor (HR) and HER2 status regardless of Ki-67 index [2], [4], [8]–[11], while a small portion of literature categorized according to the St. Gallen 2011 consensus criteria [7]. Besides, prognostic patterns of pT1a-bN0M0 patients with HER2-positive tumors varied by study even under identical definitions of intrinsic subtypes. Gonzalez-Augulo and colleagues demonstrated that a higher risk of recurrence arose in HER2-positive (regardless of HR status) patients without trastuzumab than that in triple-negative and HR-positive/HER2-negative women [4]. By contrast, Livi's report failed to discern the significant difference in disease-free survival (DFS) among the three groups [2]. Therefore, it is necessary for further investigation on the prognostic role of intrinsic subtypes in pT1a-bN0M0 breast cancer.

HER2 positivity, a known risk factor for recurrence and mortality, also serves to guide adjuvant trastuzumab therapy. Several large multicenter clinical trials have unequivocally revealed the striking effect of adjuvant trastuzumab on the improvement of prognosis for HER2-positive breast cancer [12]–[15]. Furthermore, a recent meta-analysis of published randomized controlled trials verified these impressive results [16]. Nonetheless, pT1a-bN0M0 population was scarcely included in these trials. The 2012 updates to the National Comprehensive Cancer Network® (NCCN) Clinical Practice Guidelines in Oncology for Breast Cancer [17] as well as the St. Gallen International Expert Consensus on the Primary Therapy of Early Breast Cancer 2013 [18] have both included a recommendation for consideration of adjuvant trastuzumab in pT1bN0M0 rather than pT1aN0M0 HER2-positive women, while the European Society for Medical Oncology (ESMO) Clinical Practice Guidelines 2011 [19] held that the use of trastuzumab should be discussed with HER2-positive pT1a-bN0M0 patients. Nowadays, a few studies have been conducted to assess the effect of adjuvant trastuzumab in pT1a-bN0M0 patients with HER2-positive tumors [20]–[24]. However, all these studies were limited by small sample size and retrospective nature. Therefore, insufficient evidence is available to support the use of adjuvant trastuzumab in this setting, which makes it obliged to obtain the timely insight into the appropriate management of these patients.

On these premises, a meta-analysis was carried out in two parts for pT1a-bN0M0 breast cancer patients: one was to evaluate the prognostic profiles of intrinsic subtypes; the other to explore the survival benefits of adjuvant trastuzuamb in HER2-positive phenotype. This analysis sought to provide the implications for the underlying distinction in tumor biology between different subtypes as well as for the paradigm shift in how we deal with HER2-positive population in pT1a-bN0M0 women.

## Methods

### Search strategy

The selection of publications for inclusion were performed independently by two of the authors (Qiong Zhou and Wenjin Yin), with the last search on 10 September, 2012. A computerized search was performed through the PubMed database (from 1966 to the present), the online proceedings of the ASCO Annual Meetings (years 2007–2012), the online proceedings of the San Antonio Breast Cancer Symposium (years 2006–2011), and the CD proceedings of the International St. Gallen Breast Cancer Conference (years 2007–2011), using the following search keywords: “small” or “T1a” or “T1b”, “N0” or “node negative”, “trastuzumab” or “Herceptin”, and “breast cancer”. Manual searches were done by reviewing the reference lists of retrieved studies, textbooks and review articles to identify additional potentially eligible studies. The language of publication was restricted to English. Letters to the editor, reviews, research protocol articles, articles based on guidelines and articles published in a book or papers published in non-English language were all excluded.

### Eligibility criteria

This analysis included all studies that evaluated the prognostic effect of different intrinsic subtypes or the administration of adjuvant trastuzumab for HER2-positive phenotype in pT1a-bN0M0 patients. For lack of prospective randomized trials, all the eligible studies were retrospective non-randomized controlled ones in nature. In these studies, patients diagnosed as invasive breast cancer with ≤1cm in greatest dimension and lymph node negative (pT1a–bN0M0) tumors were identified, regardless of the definition for intrinsic subtypes. The schedule and duration of trastuzumab administration was not considered for study selection. Trials were required not to assess neoadjuvant or salvage trastuzumab. Studies which enrolled patients for treatment with other anti-HER2 therapies were excluded. Studies presented in abstract form were considered eligible only if they elucidated the latest available data on at least one of the endpoints in this meta-analysis. In case of multiple results on the same cohort, the one with the longest follow-up interval was applied to the calculations.

### Data extraction

The following information was extracted from each publication: journal name, year of publication, first author, number of patients analyzed per subgroup, and number of endpoint events per subgroup. In the present analysis, two of the authors (Qiong Zhou and Wenjin Yin) independently extracted the information from each eligible publication. All the relevant data were further reviewed by a third investigator (Jinsong Lu) to reach consensus. No authors of original publications were contacted for verification or clarification of their data.

### Study endpoints

In this meta-analysis, the primary endpoint was DFS, defined as the time from the date of diagnosis to the date of first disease relapse or death from any cause without documentation of a cancer-related event. Secondary endpoints included overall survival (OS), locoregional relapse-free survival (LRFS), and distant disease-free survival (DDFS), defined as the time from the data of diagnosis to the date of death from any cause, the date of first locoregional relapse, and the date of first distant metastasis, respectively.

### Statistical analysis

Risk ratios (RRs) with their 95% confidence intervals (CIs) were calculated from the number of outcome events per subgroup to estimate the association of different intrinsic subtypes and adjuvant trastuzumab administration with various survival outcomes. The heterogeneity of the study results was assessed by Cochran chi-square Q statistics and I-square test, which determined the use of either fixed-effects (Mantel-Haenszel method) or random-effects (DerSimonian and Laird method) model. Since Cochran chi-square Q statistics can suffer from low power when the number of studies is low or excessive power when the number of studies is large, we also incorporated the I-square statistic, which quantifies the percentage of variation attributable to heterogeneity and is easily interpretable. Heterogeneity was considered as either a *p*-value <0.05 or I-square >50% [25].

Funnel plots and Begg's test were used to evaluate the possible publication bias regarding each study outcome. Sensitivity analyses were conducted to assess the influence of specific studies on the combined effect. All tests were two-sided and *p*<0.05 was considered significant. All statistical analyses were performed with Stata statistical software package (release 12.0; Stata Corporation, College Station, Texas, USA).

## Results

### Characteristics of eligible studies

In the present analysis, intrinsic subtypes were categorized into the three subgroups according to the different combinations of HER2 and HR status as follows: HER2-positive (regardless of HR status; abbreviated to HER2+), HR-positive and HER2-negative (HR+/HER2-), and triple negative breast cancer (HR-/HER2-; abbreviated to TNBC).

Based on the search strategy, eight eligible studies were identified and included in our analysis [2], [3], [8], [10], [20]–[23]. Four of these studies were used to assess the prognosis of different subtypes in pT1a-bN0M0 breast cancer patients, with 351 HER2+,1852 HR+/HER2-, 313 triple negative [2], [3], [8], [10]. Correspondingly, the others were obtained to evaluate the efficacy of adjuvant trastuzumab in pT1a-bN0M0 women with HER2+ tumors, including a total number of 229 women receiving trastuzumab and 261 women as control [20]–[23]. Table 1 and Table 2 show the characteristics of all the eligible studies in detail.

**Table 1:** Characteristics of eligible studies for prognostic analysis of different intrinsic subtypes in pT1a-bN0M0 breast cancer patients.

Author	Publication Year	Median/Mean Follow-up Interval	Study Endpoint	Number of Events / Number of Patients		
				HR+/HER2-	TNBC	HER2+
Curigliano G, et al.[3]	2009	4·6 years	DFS	2/158	5/71	11/150
			OS	1/158	0/71	4/150
			LRFS	1/158	4/71	4/150
			DDFS	1/158	0/71	3/150
Theriault RL, et al.[10]	2011	-	DFS	65/771	38/143	32/98
			OS	-	-	-
			LRFS	-	-	-
			DDFS	31/771	12/143	15/98
Arma S, et al.[8]	2010	1,015 days	DFS	4/364	3/29	2/28
			OS	23/364	2/29	0/28
			LRFS	-	-	-
			DDFS	-	-	-
Livi L, et al.[2]	2012	4·9 years	DFS	18/559	5/70	4/75
			OS	11/559	3/70	3/75
			LRFS	7/559	1/70	1/75
			DDFS	5/559	2/70	3/75

Abbreviations: HR+/HER2-, hormone receptor-positive/human epidermal growth factor receptor 2-negative; TNBC, triple negative breast cancer; HER2+, human epidermal growth factor receptor 2-positive (regardless of HR status); DFS, disease-free survival; OS, overall survival; LRFS, locoregional relapse-free survival; DDFS, distant disease-free survival.

**Table 2:** Characteristics of eligible studies for efficacy analysis of adjuvant trastuzumab in pT1a-bN0M0 breast cancer patients with HER2-positive tumors.

Author	Publication Year	Median/Mean Follow-up Interval	Study Endpoint	Number of Events / Number of Patients		
				No trasutuzmab	Trastuzumab	
McAuthur HL, et al.[20]	2011	6·8 years	DFS	9/45	2/54	
			OS	1/45	1/54	
			LRFS	3/45	1/54	
			DDFS	1/45	0/54	
Rodrigues MJ, et al.[21]	2010	29 months	DFS	5/56	0/41	
			OS	1/56	0/41	
			LRFS	0/56	0/41	
			DDFS	4/56	0/41	
Horio A, et al.[22]	2012	4·3 years	DFS	3/37	1/5	
			OS	0/37	0/5	
			LRFS	2/37	1/5	
			DDFS	1/37	0/5	
Frenel JS, et al.[23]	2012	44 months	DFS	13/123	2/129	
			OS	-	-	
			LRFS	-	-	
			DDFS	-	-	

Abbreviations: HR+/HER2-, hormone receptor-positive/human epidermal growth factor receptor 2-negative; TNBC, triple negative breast cancer; HER2+, human epidermal growth factor receptor 2-positive (regardless of HR status); DFS, disease-free survival; OS, overall survival; LRFS, locoregional relapse-free survival; DDFS, distant disease-free survival.

### Prognosis patterns of different intrinsic subtypes in pT1a-bN0M0 breast cancer patients

**Figure 1:**
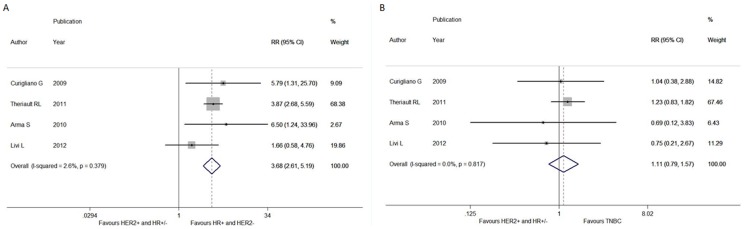
Forest plots of RRs for the association between different intrinsic subtypes and disease-free survival in pT1a-bN0M0 breast cancer patients. The size of the square box is proportional to the weight that each study contributes in the meta-analysis. The overall estimate and confidence interval are marked by a diamond. Symbols on the right of the solid line indicate RR>1 and symbols on the left of the solid line indicate RR<1. All the combined RR is calculated by the fixed-effects model. **(A) Comparing HER2+ and HR+/HER2- breast cancer. (B) Comparing HER2+ and triple negative breast cancer.**

**Figure 2:**
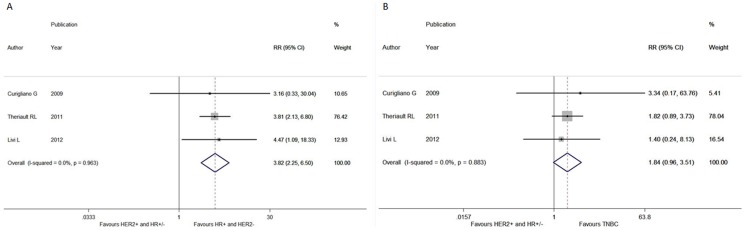
Forest plots of RRs for the association between different intrinsic subtypes and distant disease-free survival in pT1a-bN0M0 breast cancer patients. The size of the square box is proportional to the weight that each study contributes in the meta-analysis. The overall estimate and confidence interval are marked by a diamond. Symbols on the right of the solid line indicate RR>1 and symbols on the left of the solid line indicate RR<1. All the combined RR is calculated by the fixed-effects model. **(A) Comparing HER2+ and HR+/HER2- breast cancer. (B) Comparing HER2+ and triple negative breast cancer.**

In terms of the comparison between HER2+ and triple negative subgroups, significant difference between-study heterogeneity failed to be observed in the RRs for DFS (heterogeneity chi-squared, 0.94; I-squared, 0.0%; *p* = 0.817), OS (heterogeneity chi-squared, 2.04; I-squared, 1.9%; *p* = 0.361), LRFS (heterogeneity chi-squared, 0.19; I-squared, 0.0%; *p* = 0.664) and DDFS (heterogeneity chi-squared, 0.25; I-squared, 0.0%; *p* = 0.883). Through the fixed-effects model, we demonstrated that HER2+ patients elicited no benefit in DFS (RR, 1.112; 95% CI, 0.786-1.574; *p* = 0.549; Fig. 1B), OS (RR, 1.012; 95% CI, 0.328-3.119; *p* = 0.984), LRFS (RR, 0.547; 95% CI, 0.164-1.827; *p* = 0.327) and DDFS (RR, 1.836; 95% CI, 0.960-3.509; *p* = 0.066; Fig. 2B) when compared to TNBC counterparts.

When it came to the comparison of HER2+ women with HR+/HER2- ones, there was no between-study heterogeneity in the RRs for DFS (heterogeneity chi-squared, 3.08; I-squared, 2.6%; *p* = 0.379), OS (heterogeneity chi-squared, 2.60; I-squared, 23.2%; *p* = 0.272), LRFS (heterogeneity chi-squared, 0.81; I-squared, 0.0%; *p* = 0.368) and DDFS (heterogeneity chi-squared, 0.08; I-squared, 0.0%; *p* = 0.963), so the fixed-effects model was used to analyze the data and revealed a deleterious effect of HER2+ phenotype on DFS (RR, 3.677; 95% CI, 2.606–5.189; *p*<0.001; Fig. 1A) and DDFS (RR, 3.824; 95% CI, 2.249–6.501; *p*<0.001; Fig. 2A) as compared to HR+/HER2- subgroup. By contrast, no significant difference was achieved in OS (RR, 1.467; 95% CI, 0.568–3.787; *p* = 0.429) and LRFS (RR, 2.231; 95% CI, 0.533–9.341; *p* = 0.272) between these two subgroups.

### Efficacy of trastuzumab in pT1a-bN0M0 breast cancer patients with HER2-positive tumors

**Figure 3:**
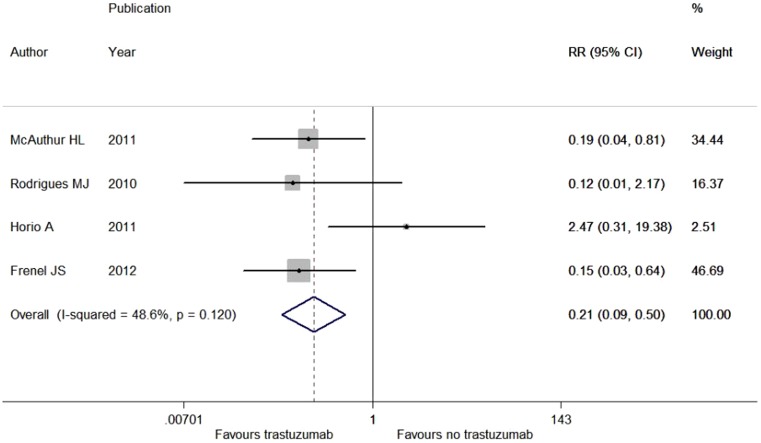
Forest plots of RR for the association between trastuzumab administration and disease-free survival in HER2-positive pT1a-bN0M0 breast cancer patients. The size of the square box is proportional to the weight that each study contributes in the meta-analysis. The overall estimate and confidence interval are marked by a diamond. Symbols on the right of the solid line indicate RR>1 and symbols on the left of the solid line indicate RR<1. All the combined RR is calculated by the fixed-effects model.

There was significant between-study heterogeneity in the RRs for LRFS (heterogeneity chi-squared, 2.82; I-squared, 64.5%; *p* = 0.093) rather than for DFS (heterogeneity chi-squared, 5.83; I-squared, 48.6%; *p* = 0.120), OS (heterogeneity chi-squared, 0.08; I-squared, 0.0%; *p* = 0.775) and DDFS (heterogeneity chi-squared, 1.70; I-squared, 0.0%; *p* = 0.428). By virtue of the fixed-effects model, we found a marked improvement in DFS with the addition of trastuzumab (RR, 0.323; 95% CI, 0.191–0.547; *p*<0.001; Fig. 3), while adjuvant trastuzumab failed to lower the risk of mortality (RR, 0.628; 95% CI, 0.080–4.916; *p* = 0.658) and distant recurrence (RR, 0.323; 95% CI, 0.064–1.640; *p* = 0.173) for HER2-positive pT1a-bN0M0 patients. Additionally, adjuvant trastuzuamb saw little effect on locoregional relapse (RR, 1.017; 95% CI, 0.073-14.187; *p* = 0.990) through the random-effects model.

### Publication bias and sensitivity analyses

For the studies regarding the prognosis of intrinsic subtypes and the efficacy of trastuzumab for HER2+ subgroup in pT1a-bN0M0 breast cancer patients, either graphic inspection of funnel plots or quantitative evaluation from Begg's test indicated the absence of publication bias in DFS, OS, LRFS and DDFS (data not shown). The sensitivity analyses clarified for both studies that no individual study affected the overall RR for DFS, OS, LRFS and DDFS, since omission of any single study made no material difference.

## Discussion

This analysis is, to the best of our knowledge, the largest and latest meta-analysis focusing on pT1a-bN0M0 breast cancer patients for the prognostic profiles of various intrinsic subtypes and survival benefits of HER2-positive women receiving adjuvant trastuzumab. Due to the rapid spreading of mammography screening and the increasing awareness of breast cancer, a growing number of women have been diagnosed with pT1a-bN0M0 breast cancers, which sequentially prompts further investigation for the prognosis and treatment of such very early stage disease.

Manifold studies have verified that intrinsic subtypes are efficient in defining prognosis for breast cancer patients [26]–[29]. However, its prognostic value is still in the air for women with pT1a-bN0M0 tumors. The present meta-analysis showed that HER2+ patients exhibited worse DFS and DDFS compared with HR+/HER2- counterparts, while the survival outcomes were comparable between HER2+ and triple negative phenotype. Our findings indicate that intrinsic subtypes might also serve as equally significant prognosticators in pT1a-bN0M0 breast cancer, just as in “large tumors” whose maximum dimensions are greater than 1cm. From this view, the biology of pT1a-bN0M0 population seems to lie more on intrinsic subtypes than on tumor size, which accords in nature with that of “large tumors”. Therefore, it is necessary for us to give priority to intrinsic subtypes rather than tumor size when tailoring proper adjuvant treatment for HER2-positive pT1a-bN0M0 breast cancer.

Besides, our meta-analysis was the first to provide substantial evidence for the beneficial effect of trastuzumab in HER2-positive pT1a-bN0M0 women with regard to DFS, which suggests that similar strategy for the targeted therapy of HER2-positive breast cancer might also be apt for such patients. Furthermore, this study provides partial support for the latest NCCN guidelines involving the recommended use of adjuvant trastuzumab in HER2-positive pT1bN0M0 women [17].

On the other hand, only the BCIRG (Breast Cancer International Research Group) 006 study [24] recruited HER2-positive pT1a-b patients among all the randomized clinical trials of adjuvant trastuzumab. However, the BCIRG 006 study was identified ineligible in our meta-analysis due to its inclusion criteria irrespective of nodal status. We performed an exploratory meta-analysis by integrating the BCIRG006 trial with the four identified studies in this analysis, which revealed a marked improvement in DFS with the addition of trastuzumab (data not shown). From this evidence, it follows that the efficacy of adjuvant trastuzumab might not vary widely by nodal status in pT1a-b breast cancer patients.

Nevertheless, there were some limitations for this meta-analysis. Firstly, all the data was drawn from non-randomized retrospective studies. Secondly, the number of cases included in our study was relatively small. Last but not least, comparison of pT1aN0M0 with pT1bN0M0 patients was not performed in this meta-analysis for lack of data. It still remains open to investigation whether the efficacy of trastuzumab varies between these two subgroups, although its use is recommended in HER2-positive pT1bN0M0 women according to the latest NCCN guidelines. Any of these limitations probably has an effect on the final results.

Even though the results of our study demonstrated that HER2-positive pT1a-bN0M0 breast cancer might have a worse clinical history and the addition of trastuzumab to adjuvant treatment could improve the prognosis of such patients, the real association between different subtypes and survival outcomes as well as the efficacy of trastuzumab offered to HER2-postive patients in pT1a-bN0M0 breast cancer is far from clear. Further studies, especially prospective randomized trials, are needed to provide sufficient data for in-depth evaluation.

In conclusion, this meta-analysis indicates that intrinsic subtypes might be a reliable marker to predict the prognosis in pT1a-bN0M0 breast cancer. Besides, even for such early stage HER2-positive patients, adjuvant trastuzumab might bring significant survival benefit. However, additional studies are still prompted for credibility.

## Supporting Information

Checklist S1 **Prisma Checklist.**
(DOC)
